# Magnetic Appendix: An Uncommon Indication for Appendectomy

**DOI:** 10.7759/cureus.31096

**Published:** 2022-11-04

**Authors:** Megan Birkhold, Joseph R Habib, Juhye Kang, Lina Diaz-Calderon, Kimberly Lumpkins, Eric Strauch

**Affiliations:** 1 Surgery, University of Maryland Medical Center, Baltimore, USA; 2 Pediatric Gastroenterology and Nutrition, University of Maryland Medical Center, Baltimore, USA

**Keywords:** multiple magnet ingestion, colonoscopy, appendectomy, foreign object ingestion, pediatrics

## Abstract

Foreign object ingestions are a common occurrence in pediatrics, often necessitating endoscopic or surgical intervention. The ingestion of multiple magnets poses an increased risk for serious complications. Our article presents a case of a five-year-old boy who swallowed two pennies and four magnets. The latter failed to pass spontaneously and were lodged in the appendiceal orifice resulting in a challenging and unsuccessful endoscopic retrieval and hence required laparoscopic exploration, appendectomy, and partial cecal resection.

## Introduction

In the United States, the incidence of foreign object ingestion (FOI) is greater than 100,000 cases per year and is a common occurrence in the pediatric population [1]. Most foreign bodies pass spontaneously through the gastrointestinal tract without complication. However, it is estimated that 10-20% of FOIs require endoscopic intervention, while as few as one in every hundred may necessitate surgical extraction [2]. Specifically, ingestion of multiple magnets entails the increased potential for enteroenteric fistulae and perforation [1]. Here, we present a case of multiple FOIs, which required surgical intervention for removal after incomplete endoscopic removal.

## Case presentation

A five-year-old developmentally normal boy without significant past medical history initially presented to his pediatrician after ingestion of two pennies. The patient’s mother was counseled to monitor for the pennies to pass in subsequent bowel movements. Five days later, the patient presented to the emergency department (ED) when four small circular magnets were missing from a playset the child had access to, without the passage of the two pennies.

**Figure 1:**
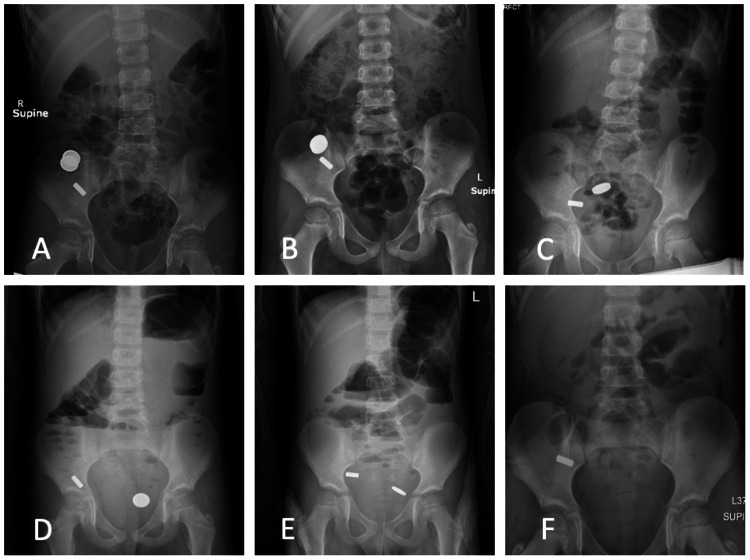
Progression through the gastrointestinal tract of ingested pennies (two) and inability to pass magnets (four) viewed via serial abdominal X-rays

Upon presentation to the ED, labs were within normal limits, the patient was clinically asymptomatic, and he denied nausea, vomiting, constipation, abdominal pain, or difficulty breathing. An abdominal X-ray (AXR) obtained noted the metallic foreign material in the right lower quadrant (RLQ), without evidence of free air and a non-obstructive bowel gas pattern (Figure 1A). He was started on an aggressive bowel regimen including MiraLAX, Dulcolax, and enemas with plans for serial AXRs. He was subsequently transferred to our medical center for further care after failing to pass the ingested foreign objects despite having multiple bowel movements. Figures 1A-1F display serial AXRs showing the progression of pennies distally through the gastrointestinal tract, presumably through the colon, with the magnets noted to be stationary in the RLQ.

Colonoscopy

**Figure 2:**
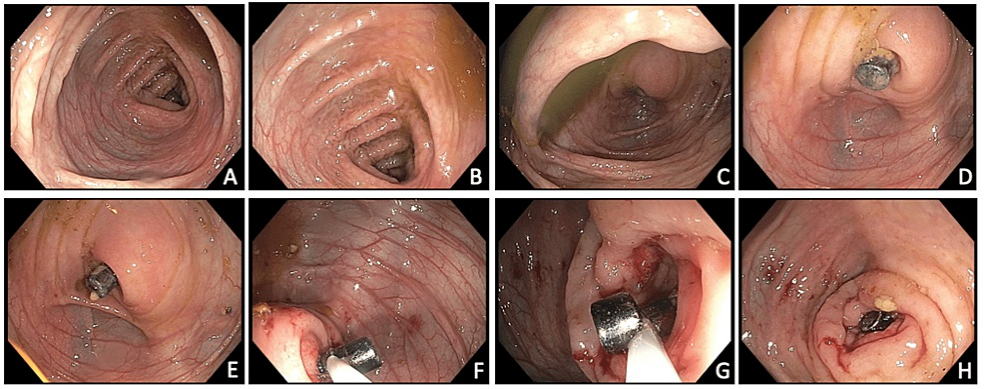
Colonoscopy images: (A) transverse colon, (B) ascending colon, (C) cecum, (D-E) appendiceal orifice, and (F-H) unsuccessful attempted removal of magnets with snare

Due to failure to pass the ingested magnets by day two of the bowel cleanout at our hospital, the child underwent a colonoscopy for endoscopic removal. The bowel preparation was excellent. Upon ileocecal intubation, the stack of round magnets was found lodged in the appendiceal orifice (Figure 2). Multiple removal attempts were made, employing a Roth Net retriever, snare (10 and 13 mm), and rat tooth device unsuccessfully. It was then noted that there was erosion into the surrounding mucosa after multiple extraction attempts and manipulation.

Operation

**Figure 3:**
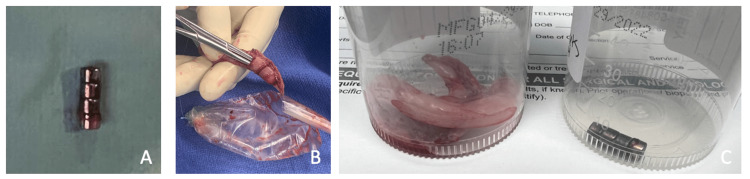
(A) Four ingested stacked magnets after removal. (B) Noted perforation where magnets were extracted. (C) Specimens including appendiceal-cecal junction and magnets

After the unsuccessful retrieval during colonoscopy, the child was taken to the operating room (OR) for laparoscopic exploration. In the OR, the patient was noted to be distended; however, this was initially thought to be due to the insufflation from the extended colonoscopy. An infraumbilical incision was made for the 12 mm port, and upon entry through the fascia, a significant amount of extra-luminal air was noted. The 12 mm port was placed under direct vision followed by a left lower quadrant and pelvic 5 mm ports. The instruments immediately gravitated toward the appendix due to strong magnetic attraction. The cecum was mobilized from the retroperitoneum, and the magnets were found to be partially extruding at the appendiceal-cecal junction. There were four magnets stacked together, and these were removed from the perforated appendix (Figure 3). The appendix was significantly injured by the magnets with possible perforation from manipulation during the colonoscopic attempted removal. The mesoappendix and the base of the cecum were then mobilized and resected utilizing the Endo GIA™ blue load stapler (Medtronic, Minneapolis, MN) to include the perforated segment. There was minimal contamination from the perforation because the patient had an excellent bowel prep.

Postoperative course

**Figure 4:**
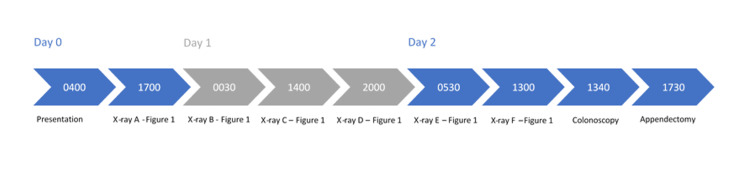
Timeline of events: presentation, x-rays, colonoscopy, and surgery

The child was observed overnight. He tolerated a regular diet and was discharged on a short course of oral antibiotics the next morning. He was seen in the clinic a few weeks after discharge and was doing well. Figure 4 displays the timeline of events from the presentation to our emergency department to surgery.

## Discussion

FOIs commonly occur in the pediatric population and may lead to serious gastrointestinal complications. Coins are the most frequently ingested foreign body; however, a variety of objects are commonly ingested, including magnets, buttons, small toys, batteries, and erasers [3]. The ingestion of two or more magnetic objects specifically has been associated with bowel wall erosion and necrosis, resulting in intestinal perforation [4]. Thankfully, in our presented case of multiple ingested foreign objects, the ingested pennies were not magnetic thus permitting an initial period of conservative management.

The current guidelines for the management of FOIs in children remain largely investigational and clinical decisions are made on a case-by-case basis. According to one center that followed four patients with magnet ingestion, medical management with daily imaging for a few days was recommended, followed by exploratory laparoscopy if imaging revealed a gap between magnets, lack of migration within 48 hours, or if the patient clinically deteriorates [5]. Another retrospective analysis of 49 pediatric patients who swallowed magnetic buckyballs recommended gastroscopy for patients with foreign bodies present in the stomach or esophagus, and emergency surgery for symptomatic patients and those who fail conservative treatment [6]. A management algorithm for magnet ingestion was developed by Sola et al., suggesting the highest risk existed in patients who swallowed multiple magnetic objects [7]. Overall, consensus exists in the literature that once the FOI is impacted in the appendix, active management with endoscopic retrieval or surgery should be pursued [8].

Previous case reports of appendiceal foreign bodies in children were most often due to ingestion of thin, sharp objects such as needles or screws with presenting symptoms of acute appendicitis, including abdominal pain, vomiting, anorexia, and fevers [9,10]. Nazzal et al. presented a case of a 23-month-old female presenting with decreased activity and continuous vomiting, who ingested multiple magnets that became lodged in her appendix. The patient was managed by elective colonoscopic removal of the magnets, which proved to be an effective and safe option [8]. Our patient failed conservative management and underwent colonoscopy followed by laparoscopic intervention for successful removal of the foreign objects. Laparoscopic removal of appendiceal magnetic foreign bodies was reported in the case of two pediatric patients who had swallowed magnetic beads, following the failure of endoscopic retrieval [11]. The foreign objects were successfully localized in the bowels using the magnetic forces between the beads and the laparoscopic tools as was demonstrated with our patient.

## Conclusions

FOIs remain common in the pediatric population and ingestion of multiple magnets poses a potential for more significant complications. The lack of symptomatology in patients emphasizes the importance of heightened caregiver awareness and safety precautions to prevent FOIs and the need for careful in-hospital monitoring of patients. This report highlights the necessity of case-by-case analysis of each patient, as well as flexibility among treatment teams to successfully mitigate the possible harms of FOIs in children, as initial attempts for treatment including medical management and endoscopic retrieval may not always be successful.
